# The Uses of Hypnotism in Medicine

**Published:** 1893-05

**Authors:** Hamilton Osgood

**Affiliations:** Boston, Mass.


### THE USES OF HYPNOTISM IN MEDICINE.[1]

BY HAMILTON OSGOOD, M.D., BOSTON, MASS.

I HAVE been asked to speak on "The Uses of Hypnotism in Medicine." If I had read the proof of the card of invitation, I should have substituted for the word "hypnotism" the words "hypnotic suggestion," or perhaps the word "suggestion" alone.

There is a good deal of curious misinformation afloat in regard

---

[1] A smoke-talk (extempore) before the Harvard Odontological Society, October 27, 1892.

to hypnotism. In the first place, in effecting cures hypnotism plays a very secondary part. Suggestion is the main influence and wholly dominates hypnotism. Charcot, who has been a prominent investigator of this subject, does not agree with this; but although he has done much to bring this matter before the scientific public, he is very full of failures, and his theories have been exploded by scientific men throughout the world; and Bernheim and Delbœuf, members of the Psychological Congress in London, at which I was present in July last, went so far as to assert that there is no such thing as hypnotism,—but only suggestibility.

You can hardly get a fair idea of this subject without learning something of its history and growth; but I will first say that, having explained my meaning with regard to the word hypnotism, I shall continue to use that word this evening because it is in common use.

Auto-hypnotism, or self-hypnotism, was used by the Magi of Persia and the Jogi of India twenty-five hundred years ago. These men threw themselves into trances by fixing the eyes intently on the end of the nose. The powers of the magnet have also been known and valued from the days of antiquity. Then we come down to the middle of the sixteenth century, when animal magnetism was first mentioned. Paracelsus was the first man to write on this subject, and his theories were studied very thoroughly by Mesmer, who, in 1779, came out in a useless book in which he announced a new treatment which he called "animal magnetism," but because of Mesmer's great success the name of the treatment was very quickly changed to "mesmerism," and if you wish to read about the influence of the *baquets* which Mesmer constructed, and about the tanks of water, the chains, and other things which he magnetized, in order to treat many people at once, you can find an account of the matter in Dumas's "The Queen's Necklace."

Mesmer believed that in certain gifted individuals there resided a fluid analogous to that found in the magnet, and that this fluid could be propagated upon other individuals by the operator at will; and in that way he suggested his cures. He had a large following, and made a great deal of money, and the attention which his treatment attracted led the French Academy to inquire into the subject; but it was abandoned by this body as not being proved and not being useful. Fix in your minds, then, that hypnotism or mesmerism is the same thing as animal magnetism. Animal magnetism is what Mesmer used, or what he taught, but simply because he was the first to practise it it was called "mesmerism."

After Mesmer, the Marquis de Puységur also treated the sick by the use of magnetism, and had so large a following that he could not attend to his patients. He therefore magnetized a large tree in front of his house and also glass rods and bowls of water, and his patients went to these various things and got their cures.

Then there is a gap, and for a number of years the matter was left principally in the hands of charlatans, with occasional interest on the part of scientific men. In 1814 a Portuguese, born in India, the Abbe Faria, gave exhibitions of magnetism in France and in England. He was the first to assert that whatever the effect might be, it did not proceed from the operator, but took place in the mind and body of the subject. Mr. Braid, a surgeon of Manchester, England, in regular standing, attended Faria's exhibitions. He became interested; he began to think the matter over, and finally took it up himself, and in his book gives a record of sixty-eight cases which were successfully treated. He also asserted that the whole thing depended upon the patient,—that if the patient's mind did not respond, no effect followed. The method he employed was to have the patient look at some bright object, usually his lancet-case. This intense fixation of the eyes frequently produced sleep. Charcot strikes a gong or flashes a bright light, and his patients, who are always hysterical or hystero-epileptics, are instantly put to sleep. These methods, such as the use of a sudden light, striking a gong, or fixing the eyes intently upon a very bright object, produce sleep; but it is a pathological sleep produced artificially by these methods, which are more or less injurious. The school of Nancy uses simply suggestion, which I will explain a little later.

Braid's influence led many scientific men, notably students of psychology in England, to study the subject, and about that time a man by the name of Grimes introduced the matter into this country under the name of electro-biology, and he had rather a large clientele. It was first applied to dentistry by a Boston man. I have never been able to learn his name, but I found the fact recorded somewhere, and used it in my first paper on the subject, some three years ago, after reading which a man called on me and, if I remember rightly, told me he was the dentist in question, but I was busy just then, and did not have time to listen to what he had to say.

After Braid there followed another hiatus, until we come to the year 1866, when Liébeault published a book on "Suggestive Therapeutics." That book was the result of years of practice, and he was the first man to use suggestion exclusively,—that is, he was the first man to talk to his patients. He was called erratic and the book was shelved. In 1878 Charcot began his experiments; but we can dismiss Charcot by saying simply that his theories are full of error, while yet admitting that he has done a great deal to put the subject on a scientific ground. In his writings he says that he thinks suggestion useless.

In 1882, Professor Bernheim, of Nancy, who is the head of the medical school in that city, was asked to go and see Liébeault, to whom I have referred. Bernheim at that time was a thorough sceptic in regard to this subject. He said it was all nonsense, and that he would not waste time going to see Liébeault's work. A friend of his finally almost dragged Bernheim into one of Liébeault's clinics, and what he then saw so astounded him that he immediately began to investigate the matter by applying the treatment to his hospital patients, and two years later published a book entitled "Suggestion," which, by the way, is one of the best books written upon this subject, and can be obtained in this country in translation. Then Beaunis, another professor in the same city, took up the physiological bearings of suggestion, he published a book, and so the thing steadily grew, until to-day the literature of the subject is very large; there are two journals regularly published in the interests of hypnotism; there is a hypnological society, which meets every month in Paris; and I had the pleasure of attending one of its meetings in July last.

As a tribute to his memory, I will remind you that Liébeault, whose book was derided in 1866, is now considered the father of the modern school of hypnotic suggestion. He practised the treatment nearly thirty years, almost without remuneration, his patients being poor in possessions, but amounting to many thousands in number. The actual head of the school to-day is the brilliant Bernheim, of Nancy. Wetterstrand, of Stockholm, Forel, of Zürich, Moll, of Germany, Van Eeden, of Holland, Bérillon, of Paris, Lloyd Tuckey, of London, are well-known hypnotizers. They have all published books on the subject, these books being but a few of the large number now in print, and written by medical men of various countries.

With reference to the history of hypnotism, I think I have mentioned all that one needs to know; but I wish to impress upon your minds that animal magnetism is dead; there is no such thing as the passage of fluid from one person to another,—that theory is all gone.

Hypnotism can be very wrongly used, and it is very much misunderstood. In comparison with the proper means of putting a person to sleep, hypnotism is not only unnecessary but it is objectionable. In hypnotizing a person some artificial method is used; for instance, a bright object is held before the eyes, the subject is required to fix his gaze upon it, and the muscles of the eyes being intimately connected with the brain, soon become tired. You can see, then, how, particularly in a very sensitive person, trouble would be apt to arise in the nervous system, so that often patients have been thrown into hysterical convulsions by the use of such methods, and these instances are cited by some medical men to prove that the whole system is dangerous. In one case, at an evening party, some people proposed hypnotism as an amusement. A diamond ring was held before the eyes of a young man for fifteen minutes and he finally became hypnotized, but he felt the effects of it for about three months, simply because nobody knew how to bring him out of it. That is enough to prove that to use this treatment upon anybody for amusement is wrong. I never use it for any purpose whatever except to relieve pain, to impart vigor, or to overcome a bad habit, such as intemperance or the cocaine habit. The possibility of being able to cause hallucination is a great temptation. You have a man asleep and his condition is such that he will do exactly as you direct, and it would create considerable amusement to by-standers to make him think himself an alligator, or a soldier, or a priest, or what not; but all that is not only wrong, it is wicked, it is an unjustified interference with the personal liberty of the subject. The use of suggestion should be confined strictly to alleviation of distress or stimulation of the functions of the organism of the patient.

I regret that more of our medical men are not interested in the application of this method of treatment. There are various reasons for their lukewarmness and prejudice. In the first place, it is a new thing for physicians thus to treat their patients. Secondly, the method has long been in the hands of charlatans, and for this reason it requires courage to use it in practice. Again, there is, here and there, a physician of influence who pronounces the treatment dangerous. Let me say, however, that whenever a medical man makes this statement you may be certain he is unfamiliar with the method. He may have read about it, and he may have read largely, but he also may have read for years upon four-in-hand driving, or upon navigation, but this does not make him either a good whip or a good sailor. Practical familiarity with the uses and application of hypnotic suggestion is the only title one can possess to an opinion upon it. But if a physician of influence pronounces it dangerous, the lesser lights, like sheep following a leader, jump the same wall, and therefore let the treatment alone. Dangerous! Why, morphia, strychnia, ether, are a score of times more dangerous, and we are never quite sure how they are going to act. Whereas, hypnotic suggestion applied by the verbal method is absolutely harmless, beneficent, and helpful. But I wish we might have a law forbidding all public exhibitions of "animal magnetism," so-called, because they have done a great deal of harm. The exhibitor does not see his subjects again, and does not know what the effect has been on those persons. The whole thing is wrong and an outrage, and in the track of those people who give public exhibitions there is always found more or less nervous trouble, the result of overtaxing subjects and the want of proper care.

The fact is, hypnotism should be kept wholly in the hands of medical and scientific men. In five European countries these outrageous exhibitions are forbidden by law. In this country the city of Philadelphia is the only place in which they are not allowed.

Now, I think we are ready to take up the matter itself. A person comes for relief, and he is to be relieved by suggestion. He is not put to sleep by any waving of hands or so-called mesmeric passes. The influence used is simply suggestion underneath and throughout the whole proceeding. If passes are made over a patient, and he thinks they are going to make him sleep, he will sleep; but passes are unnecessary and smack of charlatanism. No person can be put to sleep unless he knows he is going to be put to sleep and is willing; and if the physician properly addresses a patient who is perfectly willing, that patient, if he be suggestible, will receive the suggestion and sleep, and the passes are entirely unnecessary. Just now occurs to me one of the objections raised by the laity,—viz., "I would not give up my will to anybody." Now, we will say that a man has chronic trouble and has to be treated a number of times. He comes to me and receives his treatment just as if I were treating him locally for some affection of the throat. As I am doing what he wishes me to do, he does not give up his will to me, but I am serving him and rather giving up my will to him.

The patient falls into a pleasant and what appears to be a natural sleep. Then the suggestions are made. If the patient is suffering in any particular part, it is my habit to stroke that locality gently with my hand while I make verbal suggestions. This procedure seems to call nervous energy to that part by attracting the patient's mind to it. The patient is asleep, but he hears everything I say to him; he may snore, and yet the auditory nerve conveys to his mind every suggestion I may make; and if I say, "You may awake now," he will awake at once. The patient has done as I wished, his mind has received what I have said, though he may not remember a word of it after waking, and he finds he is relieved. On the other hand, the patient may be largely conscious during his somnolence, and still he can be relieved by suggestion. How does that occur? I will tell you. The upper part of the patient's brain *is* asleep; it is asleep enough to have lost its intellectual initiative. The patient of himself cannot initiate an idea, but still possesses what may be called sub-consciousness. This is what Holmes calls "the other fellow." It is our automatic self. It is that state of mind which impels you to step over a hole in the sidewalk, or get out of the way of a team or horse-car when crossing the street, even though you are mentally unconscious of these objects and very much interested in the conversation of your friend as you walk along. But if your friend afterward ask you, "Do you remember that hole in the sidewalk which we passed a few moments ago?" You will say, "What hole? I don't remember any hole; where was it?" "It was on Boylston Street, opposite Arlington. There was a hole; it was unguarded and we had to walk around it." Then you say, "Oh, yes; I do remember, and I wondered why it was allowed to be there." This is the sort of consciousness which is active in patients who are not fully asleep,—not sufficiently so to have lost memory on waking of what has transpired during the sleep. Patients sometimes say to me, "I haven't been asleep; I heard you walk across the room and turn over your book; I kept my eyes closed merely because I supposed you wished me to do so." But the patient meanwhile has been relieved. I have actual contests with some of my patients who insist that they have not been influenced, and I have to say to them, "You will be kind enough not to have any opinion on this subject. It is impossible for you to judge. The fact that you are relieved is proof that you have been influenced." In the end patients acknowledge that they were wrong.

After a patient has come back to a thoroughly wakened state he may think that he has been intellectually awake all the time, but he has been in one of the first six of the nine stages into which Bernheim has divided the hypnotic condition. Some writers claim that there are but three different stages, but I prefer to accept Bernheim's nine stages, which are pretty sharply marked. In the first of these the patient is somnolent, but he is suggestible and can be relieved in many ways. He can open his eyes and is perfectly conscious of all that is going on. In the second stage he is more suggestible. If told that he cannot open his eyes and is asked to try to do so, he finds he cannot. In the third stage automatic obedience and suggested catalepsy begin. If the physician wish to test the patient he lifts the patient's arm and there it stays. If asked as to whether he can put it down, the patient tries and finds he can do so. In the fourth stage he cannot put the arm down when once it is raised by the doctor. He may be sufficiently conscious to say "I cannot do it," and the arm remains in that position as though carved. It is pushed down with great difficulty, and when released immediately springs back into its place. Now, what is the cause of this? That man has lost his initiative. He cannot initiate ideas of himself, and the action of the mind which keeps the arm in a certain suggested position is governed by the suggestion which it received from the operator when he said, "You cannot put down your arm." In this condition of somnolence he is unable to create the idea, "I can put it down," and thus the idea which is dominant in his mind and which controls the muscles of the arm is contained in the suggestion, "You cannot put down your arm." In the fifth and sixth stages automatic obedience increases, so that if these tests are applied, and I very rarely apply them, the patient, in obedience to suggestion, will arise, will walk, or assume a given position, and, if the operator say, "You cannot come forward," or "You cannot go backward," the patient finds he cannot do so. This is called automatic obedience.

In all these stages the patient knows more or less of what you have said to him, and after he awakes remembers either indistinctly or very distinctly what has happened. Then comes a gap,—but to be brief, in the seventh, eighth, and ninth stages, the loss of memory is complete, and the patient after he awakes knows nothing of what has happened. In those stages hallucinability begins; one can cause dreams in a patient; make him think what one pleases, and, of course, automatic obedience is more pronounced. These last stages are very seldom reached in my experience. Among the French I have seen many cases in which these conditions existed, but the patients were mostly servants, or soldiers, or people who have been accustomed to obey, or hospital patients who have been long under the influence of hypnotic suggestion. They have no fear of it, and imitation has a very great influence. One patient sees another asleep, and when the suggestion is made he goes to sleep very readily. Perhaps it was due to this imitation that Lié-

beault attained the success he did, when, out of one thousand and fourteen patients, only twenty-nine were "refractory,"—that is, failed to be influenced into one or another of the degrees of which I have spoken. That success would never have been reached in this country, for the reason that Americans are not so suggestible as the French.

Now, in what way can this treatment be used? There is hardly anything in which I would not use hypnotic suggestion. Cases of neuralgia, rheumatic pains, loss of voice, stammering, nervous prostration, fixed ideas, intemperance, the cocaine and morphia habit, functional paralysis, wry neck, and many other ailments which I might mention, have yielded to suggestion at my hands. And the treatment is direct and immediate. There are many cases, of course, which require repeated application of the suggestion, and the amount of treatment necessary always depends upon the varying susceptibility of different individuals. You may ask, How can suggestion produce a functional effect? I will try to explain: Suppose I make a very personal remark to a lady. Her cheeks will redden at once. Now, how does this blush occur? The lady knows nothing about the mechanism of blushing; she is unconscious of the process; she cannot arrest nor create it. But, I have aroused in her mind an emotion, and the effect of this emotion passes out of those cells in which mental action takes place, and runs along the intricate nerve connections which finally carry it to the spinal cord. From the cord it passes into the sympathetic nerves which supply blood-vessels with the vaso-motor nerves. Under the influence of the emotion which I have created, these nerves momentarily lose control of the blood-vessels, which dilate, and the result is a flushing of the face, caused also in part by a quicker action of the heart, which is stimulated by these same sympathetic nerves and is also affected by the temporary mental agitation of the lady I have addressed.

This is a good illustration of the manner in which the heart is reached by hypnotic suggestion, and the same is true of any portion of the body; so that if, for instance, I have a patient asleep who has very cold hands and I suggest that those hands become warm, they will do so in three or four minutes. I have caused the heart of a patient to beat with absolute steadiness when it was losing every sixth beat, and these illustrations of the functional effect of suggestion are very common in my practice.

I have seen what seemed to be marvellous cures of intemperance, and perhaps the worst case was that of a man who was brought to me by his sister, who told me that he had been intoxicated every night for twenty years. He was relieved of the habit so that he has felt no desire for drink for the past three years, and of thirteen cases which I have treated for intemperance, eight thus far seem successes. These cases are a proof that this treatment does not weaken the will. On the contrary, I strengthen the will of an intemperate person by suggesting to him that it will not make any difference how many men ask him to drink, that he has the strength of character to say "No," and the desired result follows.

Of course, chronic trouble requires chronic treatment,—that is an old adage. If an ailment be acute, as, for example, hoarseness, it can be relieved almost at once; and it is the same in neuralgias, rheumatisms, etc., of not long standing; but the cases which have lasted long enough to create a mental habit must be treated long enough to create another mental habit,—you have to plough another furrow over the one already existing, as it were.

With reference to the anæsthetic uses of suggestion, I may mention cases in which the introduction of the speculum was very successful, because the patients did not suffer the least pain. The patients were put to sleep; I introduced the speculum and made the necessary applications; and then aroused the sleepers and found them to be in a refreshed condition, instead of being tired, as would usually be the case. I have used suggestion in only one case of labor, and in that instance the patient was kept under the influence of suggestion for two or three hours, the uterine contractions continued as usual, and the birth would have been absolutely painless had the patient not begged me, during an interval of wakefulness, to leave her awake. I did so, but regretted it later. I have done some things with the knife, but surgery not being my specialty, I have had to wait for my cases; still I have performed some minor operations which would have been very painful, indeed, if the patient had not been under this influence. One case in particular was that of curetting an old abscess in the buttocks. The patient was in such deep anæsthesia that I could have amputated his leg without causing a particle of pain.

I am not an enthusiast in this matter,—I am simply a convert. I began to read about hypnotism some years ago; and the book I first read was on animal magnetism, by Binet and Feré, Charcot's assistants. This book was confusing and unsatisfactory, and, as I afterwards learned, was full of errors. I then went abroad, and spent two or three weeks with Bernheim and his associates, seeing things every day that seemed almost marvellous. After my return to

Boston, I began to use the treatment in cases in which it seemed better than the old remedies, and it has given me great satisfaction.

Now in regard to its use in your profession. A lady came to me who was very timid about having her teeth filled, and was very nervous; and as habit or experience, after a time, gives a physician who uses this treatment a very good idea of those persons who are likely to be suggestible, I decided at once that she was very suggestible. She said she had a tooth which needed filling badly, but that she did not dare to go to the dentist. I said to her, "Just come here and look into my eyes. Where is this tooth that is to be filled?" She told me, and I stroked the jaw a few times with my hand, and simply said, "It will not hurt you in the least to have this tooth filled." She afterwards told me that the dentist expressed surprise that she did not complain under the touch of his instrument, and that she herself thought the tooth must be dead.

You can see how very useful this treatment ought to be in your profession, and I dare say Dr. Fillebrown will tell you of many cases which will interest you. He has told me that he has kept patients in his chair for two or three hours in more or less consciousness of what he is doing and yet not suffering. Anæsthesia may exist in any one of those degrees of sleep which I have described to you. Of course, the anæsthesia is a suggested condition, and is based upon the suggestion "There will be no pain." There is one case on record of a woman who, during labor, was almost awake, and yet she was absolutely surprised when she found her child was born. She had received the application of hypnotic suggestion a number of times before her labor came on, and had become thoroughly convinced that there would be no pain. At the beginning of the labor her doctor put her into this semi-conscious condition, with the result which I have mentioned. This is a good example of anæsthesia which is as effectual as chloroform or ether, but without the bad effects usually attending the use of those anæsthetics.

Patients differ very much, indeed, in their reception of this influence. Some patients fall into a deep sleep and remain asleep during the entire time of filling or dressing a tooth, and should they become uneasy, they can be quieted by suggestions, such as "You won't awake," "You won't suffer any pain," "This will not hurt you," etc. Talk exactly as if the patient was awake; but—and this is a very large but—there is one thing that I wish to call to your attention: the men abroad who are prominent in the use of this treatment believe, as I believe, that the people who employ it should have a medical education. You cannot play with this thing; you cannot properly use it without a proper education. There are a very few cases on record in which harm seems to have been done, but simply either from ignorance or foolishness on the part of the operator. He has played with his patient and amused himself. He has a very good subject, and he creates a certain abnormal hallucination, and does so frequently, the result being that, perhaps, the patient would begin to have that same hallucination in a waking state; or, these bunglers have put their patients to sleep by the use of some bright object, straining the eyes, thus irritating the brain, and doing injury in that way. Dr. St. Clair Thompson, in an article in the *Westminster Review* on the dangers of hypnotism, says that its use has produced lethargy in some people, and the newspapers have given accounts in which patients have fallen into a state of lethargy. These have uniformly been hypnotized by laymen for amusement, and a doctor having been called, he has failed to awaken the patient, and has resorted to electricity, stimulation, or some other means than the right one, simply because he also was ignorant of the proper methods. Now these cases beautifully illustrate the power of suggestion. When a person is profoundly asleep, and he does not awake at once on being ordered to do so, the person who has hypnotized him becomes frightened,—in all these cases we have to deal with the laity, not persons who have been educated in the use of suggestion,—and the by-standers then begin to try various methods, and scream into the patient's ear; and the patient himself, whose suggestibility, I suppose, you will all understand, was normally increased by the mere act of putting him to sleep, receives the suggestion that it is very hard to wake him, and he will sleep all the more profoundly. Then they send for a doctor. The doctor doesn't know anything about the necessary technique, and he uses some absurd method which produces no effect whatever. Finally, we will suppose, a doctor comes who knows something about suggestion, and he says to the operator, "Put me into relation with this subject. Just tell him that Dr. X. is here, and that he is to listen to him." That being done, the patient will listen to him. The doctor will then proceed in a confident manner, and perhaps press the patient on the top of the head, or gently pinch his ear, at the same time saying to the by-standers, "This always wakes a person," and then sharply gives the command to "wake." The patient receives the suggestion, and he will wake at once.

The inexperienced operator in his early application of suggestion may have a lot of susceptible people at first, just as one is apt to have a run of rare cases. I remember at one time I had a series of six cases of angina pectoris, and I may never have another one,—it is not a very common disease. Well, he has a run of susceptible people, and he thinks it is all easy enough, and becomes careless, until finally he has a patient who is very nervous, and may give an inexperienced person considerable annoyance. One must adapt himself to every individual case,—cases differ. Involuntarily you treat your patients differently, according to the conditions which present themselves, and you would have to do so if you ever hypnotized; but it must be understood—and let me say that I think in any case it is a very great necessity—that every dentist should have a medical education. If I were a dentist I should be afraid all the time that someting might happen to my patient, and if a very nervous person came to have a tooth extracted, I should not know whether it were a safe thing to give that patient ether or gas; and coming back to the feeling of the teachers of this subject abroad, I feel with them that whoever uses this method of treatment should go to some one who can properly educate him in the use of suggestion, and a dentist could then use it for the purpose for which he learned it,—that is, to give his patient a comfortable time in the chair. The dentist has no business to try to relieve every trouble of which the patient may complain, any more than I have the right to try to clear out a dental cavity without proper education and experience, because, aside from mutually aiding each other, as we should, we should protect and we ought to be just to each other. When dentists do take up this treatment, they ought to be as religiously careful in its use as I am. Do not use it until you understand it; if you do, you will get into trouble; and then when you do use it, let it be simply to keep your patient in comfort. When a patient comes to me for relief from a given ailment, I confine myself to that trouble. You can imagine how useful this treatment would be to you when you are operating for one of those very nervous patients who are constantly seizing your hand and jumping and crying, "Oh, I can't bear this," and so on. If you can keep such a patient quiet, you can do more work in the same space of time and with less wear and tear on your own nerves. But, as I say, in using it, you ought to be thoroughly educated in the technicalities of suggestion, and you should have a certain amount of medical education, so that if anything should happen to the patient you could meet the trouble. One day, I was treating a patient whom I had not seen before, and she was suddenly seized by an epileptic attack. If I had not been a physician, I should have been very much alarmed, for, naturally, I should have suspected that I had caused it. The attack was a light one. I waited quietly, and it was soon over. I then said to the lady, "I suppose you often have these attacks?" and she replied, "Oh, yes, very often," so that, as I had supposed, it was nothing but a coincidence; but I should have been very uncomfortable if I had known nothing about it.

There is one thing I have not touched upon, and that is the danger. Physicians, otherwise intelligent, sometimes say, "I consider this treatment positively dangerous." Now, practically, such men know nothing about it. I have hypnotized a great many hundreds of times, and have not seen a single objectionable effect, nor do Bernheim, with his thousands of cases, and Liébeault, with at least fifteen thousand, record any instance where harm has resulted. In all these cases verbal suggestion only was used,—no artificial method. Of course, every man takes care to say to the patient while he is asleep, "You won't have any headache; you will feel refreshed after you awake," etc.; and these suggestions are answered in kind. Moll, a German, who has also written a fine book on this subject, says that this treatment is like the use of electricity, or the use of the catheter,—you have got to know how to use them. Every time a doctor gives a hypodermic injection of morphia, he is exposing his patient to more danger, a hundred times, than is the man who puts his patient to sleep, and the same thing is true of many other drugs which are used in medicine, where the fraction of one grain has been known to take life.

I don't know whether you are as frank about dentistry as some of us are about medicine, but the practice of medicine is very uncertain, and we don't know how drugs are going to act. Ninety-nine men may be helped by quinine, which is a specific for malaria, but when it comes to the one-hundredth man he is not relieved by it, and we have to try something else. Out of the long list of drugs which are used there are but very few which can be called specifics. Digitalis, we know, has a certain effect upon the heart; mercury in syphilis, and quinine in malaria. These nearly exhaust the list of specifics. The rest of our drugs are all experimental, and when Voltaire said that the practice of medicine consisted in "putting drugs of which we know little into bodies of which we know nothing," he expressed a great deal of truth.

The question has been asked me, "Is it not dangerous to let anybody hypnotize you?" I answer, yes; you must know your man; and if I were a dentist, and were going to hypnotize, I should have some one present, or have a friend of the patient come, just as if I were going to give an anæsthetic; but if you have an absolute acquaintance with the patient, and the patient prefers to be alone with you, that would be another thing.

There is very great beauty (for want of a better word) in this treatment. It is the only treatment, except surgery, which goes directly to the seat of the trouble under treatment. When we give medicine, we are simply preparing the body to return to health. But there is an enormous amount of suggestion in the medicines which are administered. The same tonic is taken for many different complaints, and when it does not appear to be doing good, something is added which changes the taste, but which does not influence the effect of the tonic in the least degree, and the patient will begin to get better at once. While I was in France, I happened to pass through the town of Lourdes, where the Virgin Mary is said to have blessed the waters, and there I saw people from all over the world who were being cured of their ailments. This was, of course, an effect of suggestion. Then there are the electro-magnetic belts and rings which people use against rheumatism, etc., their effect is purely suggestive; and it seems to me that sincere homœopathy is nothing but suggestion, because certainly the idea that the smaller the dose the greater the effect is rather a curious one. The old-fashioned charms are plainly nothing but suggestion, such as the cure for warts, etc. A man is told to take a piece of meat,—it must be beef,—and he must rub the warts with it, and he must bury it under a certain tree at a time when the moon is at a certain height. Of course, this all seems very silly, but the fact remains that the warts do disappear,—of course, again, by suggestion.

You have need to be very careful as to what suggestions you make to those patients who are very suggestible. While I was with Bernheim he gave me several illustrations of the great susceptibility of some persons in a waking state. As we were going through the wards one day, he said to a man who was quietly sitting beside his bed, "Number 9, what was the trouble you had with number 7, this morning?" The man replied, "I had no trouble with him, sir." Bernheim said, sharply, "What do you mean by denying it? I want to know what the trouble was." The man knit his brows a moment, and then suddenly said, "Well, he stole my two-sous piece and I hit him." Now, there had been no trouble at all, and it was all a fiction of the man's brain suggested by Bernheim's remarks. On another occasion, as we were passing a patient, Bernheim said to me in a voice loud enough to be heard by the patient,

"No. 10 will go down to No. 4 and steal his night-cap." After we had gone by a little distance, I looked back, and saw the man straighten up, looking somewhat in doubt, and finally get up and walk straight down to No. 4, snatch off his night-cap, cram it into his pocket, and walk back very unconcernedly. We returned, and Bernheim asked him what he had been stealing. He said, "Nothing." "Turn out your pockets," said Bernheim, and out came the night-cap. "Where did you get that night-cap?" "I don't know, sir." "Well," said Bernheim, "I will tell you; you stole that night-cap from No. 4." The man protested that he did not, and seemed much distressed, for Bernheim made him go and return the cap to No. 4, whose face wore a look of great astonishment. Now, that use of suggestion seems to me cruel, but in this case it was justifiable, for Bernheim was simply giving me illustrations of the great susceptibility of some people in the waking state. I met an old gentleman, an acquaintance, on board ship on my way home, and in the course of conversation he said, "I do not like the nights at sea: I am awfully afraid to go to sleep at night, and don't undress at all." I said, "I think that is very strange: you ought to undress and go to bed just as you do at home. The ship is always under the care of experienced persons, and there is more care used at night than there is during the day." I thought no more of it, until this person came up to me afterwards and said, "Doctor, I have been turning that matter over in my mind, and I think I'll do as you advise to-night, and see if I cannot sleep." I thought to myself, "I believe this man is very suggestible," and therefore said to him in a confident and earnest tone, "I know you will sleep to-night: I haven't the least doubt of it." The next morning he came to me and said, "Doctor, I had a magnificent night. I went to my room and undressed, just as if I were at home, and slept profoundly;" and he told his friends that Dr. Osgood had simply broken up all his fear, and that he would now be able to sleep while aboard ship. Now, here was a man who was suggestible in the waking state, and who came to me accidentally. You can easily see that individuals, who are thus suggestible in the waking state, could be wronged by persons who know enough to make evil use of their susceptibility. Fortunately, such instances are very rare. Whenever I find that a patient is very suggestible, I protect him by saying to him while he is asleep, "No other person can ever hypnotize you, without my permission," and thereafter no one but myself can influence him.

Another point is the question as to who can hypnotize and who cannot. I have learned how, and am sympathetic, patient, gentle with my patients: they feel at ease with me, and all those things help me. My patients have perfect confidence in me and trust themselves with me; but there are men who are also sympathetic, also gentle, who cannot seem to succeed in this, because they are timid, or they lack adaptability, or are very easily embarrassed by their patients.

The question is asked, Why is it that one man can hypnotize and another cannot? I will answer that question by asking another, Why is it that of two boys playing marbles, one of them can snap a marble the first time better than the other boy ever can? Why is it that of two boys one will make a good violinist and the other will not? And why is it that one man will make a good dentist, while another man cannot seem to make a success of it, try as he may? It is because he does not possess the necessary mechanical ingenuity. A lack of the power of adaptability, of natural skill, of self-poise, are probably the main reasons why a person cannot successfully hypnotize.

This subject is very large, and one thing suggests another. I have tried to make clear to you how this treatment acts and why it acts, but there are a good many things about which I have not spoken, and if any one wishes to ask me questions on any point he does not understand, I will try to explain.

